# A high-resolution cardiovascular magnetic resonance diffusion tensor map from ex-vivo C57BL/6 murine hearts

**DOI:** 10.1186/s12968-014-0077-x

**Published:** 2014-10-16

**Authors:** Stelios Angeli, Nicholas Befera, Jean-Marc Peyrat, Evan Calabrese, George Allan Johnson, Christakis Constantinides

**Affiliations:** Department of Mechanical and Manufacturing Engineering, Laboratory of Physiology and Biomedical Imaging, School of Engineering, University of Cyprus, 75 Kalipoleos Avenue, Green Park Building, Nicosia, Cyprus; Center for In Vivo Microscopy, Duke University Medical Center, Durham, NC USA; Qatar Robotic Surgery Centre, Qatar Science & Technology Park, Doha, Qatar; Chi-Biomedical Limited, 36 Parthenonos Street, Apartment 303, Strovolos, 2021 Nicosia, Cyprus

**Keywords:** Diffusion cardiovascular magnetic resonance, Cardiac, Myocyte, Tractography, Mouse, Tensor map, MRI

## Abstract

**Background:**

The complex cardiac fiber structural organization and spatial arrangement of cardiomyocytes in laminar sheetlets contributes greatly to cardiac functional and contractile ejection patterns. This study presents the first comprehensive, ultra-high resolution, fully quantitative statistical tensor map of the fixed murine heart at isotropic resolution of 43 μm using diffusion tensor (DT) cardiovascular magnetic resonance (CMR).

**Methods:**

Imaging was completed in approximately 12 hours using a six-directional encoding scheme, in five ex vivo healthy C57BL/6 mouse hearts. The tensor map constructed from this data provides an average description of the murine fiber architecture visualized with fiber tractography, and its population variability, using the latest advances in image tensor analysis and statistics.

**Results:**

Results show that non-normalized cardiac tensor maps are associated with mean fractional anisotropy of 0.25 ± 0.07 and mean diffusivity of 8.9 ± 1.6 × 10^−4^ mm^2^/s. Moreover, average mid-ventricular helical angle distributions ranged between –41 ± 3° and +52 ± 5° and were highly correlated with transmural depth, in agreement with prior published results in humans and canines. Calculated variabilities of local myocyte orientations were 2.0° and 1.4°. Laminar sheet orientation variability was found to be less stable at 2.6°. Despite such variations, the murine heart seems to be highly structured, particularly when compared to canines and humans.

**Conclusions:**

This tensor map has the potential to yield an accurate mean representation and identification of common or unique features of the cardiac myocyte architecture, to establish a baseline standard reference of DTI indices, and to improve detection of biomarkers, especially in pathological states or post-transgenetic modifications.

## Introduction

The unique myocardial architecture, comprised of laminar sheetlets with spatially varying orientation [1-3] and helical fiber tracts, account for the heart’s efficient contractile and torsional mechanical function [4,5]. In fact, evidence supports the spatial arrangement of cardiac myocytes in minimal surfaces (referred to as generalized helicoids) as an innate compensatory mechanism to optimize organ function and contractile ejection [6].

The inherent cardiac tissue structural-functional associations also underline the potential significance of myocyte diffusion tensor CMR (DTCMR) tractography [7-11] in remodeling or cellular disarray following both early and late pathological states (e.g. ischemia, cardiomyopathies) [12,13], pre- or post-cellular regenerative interventions.

While in vivo cardiac DTCMR tractography is still in the early stages of development for widespread pre-clinical [14] or clinical use [15-17], ex vivo DTCMR has been established as a useful method to identify local, transmural distributed eigenvector orientation, and to construct three-dimensional (3D) myocardial fiber maps [4,5,18-21]. Parallel to visualization of distributed eigenvector orientation, numerous concerted efforts have been made to quantitatively assess transmural helical angle (HA) distributions, fractional anisotropy (FA), and mean diffusivity (MD) in normal and pathological states. Prior ex vivo attempts span studies on humans [22-24], canines [19,25-27], goats [28], sheep [21], rabbits [21,29], rats [12,13,30], and mice [4,21,31,32].

Historically, some earlier studies validated the direct correlation of myocyte eigenvector orientations with conventional invasive histology, despite scale differences, in freshly excised [25], perfused [29], and fixed [33] myocardium. More recent high-resolution DTCMR [34] and Gd-DPTA [35] studies validated the localized orthotropic nature of sheep and rat myocardium, respectively, with histology and identified spatial variations in its micro-laminar composition and associated branching.

Nevertheless, to date, no previous studies have presented a comprehensive, high-resolution, quantitative eigenvector orientation tensor map of the ex vivo murine heart. Additionally, only one prior study [31] targeted the extensively studied and stable C57BL/6 mouse strain. That study, however, focused only on quantifying helical angles in normal and hypertrophic hearts.

This study employs 3D, microscopic, spin-echo, diffusion-weighted CMR [36,37] to construct a myofiber tensor map of five ex vivo fixed hearts, at an isotropic ultra-high resolution of 43 μm. Main contributions of this study include: a) construction of a 3D, high-resolution, quantitative tensor map of the C57BL/6 murine heart, b) quantitative assessment of the variability of FA, MD, and HA distributions, and c) identification of transmural tissue structure anisotropy and the myocardial laminar sheetlet dominance.

This tensor map will be useful in its potential to a) characterize electro-mechanical function, reflecting an unbiased mean of a test population, serving as a deformable template, and providing tensor-based normalization of study subjects/species; b) yield an accurate mean representation and identify common or unique features of myofiber architecture, c) establish a baseline standard reference of DTI indices, and d) improve the detection of FA, MD, and HA biomarkers, particularly in pathological states.

To the best of our knowledge, this is the first murine distributed eigenvector orientation map constructed at ultra-high resolution (exceeding isotropic recordings at 100 μm by [4] in mice and non-isotropic studies at resolutions ranging from 78–2000 μm in other species [12,19,23,24,26,33]. We hope that this effort will prove useful in mouse phenotyping, targeting regional cardiac function, transgenesis, and molecular imaging. The constructed murine tensor map and DTI data from this work are directly available for public use at http://www.civm.duhs.duke.edu/cardiacMRdiffusion2014 and at http://lbi-cy.com/?page_id=80.

## Methods

### Ethics approval

All experimental procedures on mice were designed to minimize or avoid unnecessary pain or discomfort inflicted on the animals. All protocols were approved by the Duke University Institutional Animal Care and Use Committee (IACUC), in accordance with the American Physiological Society’s Guiding Principles for the Care and Use of Animals, European Animal Research directives, and International Guidelines for Animal Research.

### Heart excision and sample preparation-fixation

Male C57BL/6 mice (n = 5, ages 8–12 weeks) were anesthetized via injection of intra-peritoneal ketamine/xylazine. Endotracheal intubation was performed with trans-tracheal illumination using a 22 G angio-catheter as endotracheal tube. Mice were then maintained under general anesthesia using 1.5% isoflurane in mixed gas, delivered via a custom rodent ventilator with a tidal volume of 100 μL at a rate of 110 breaths per minute. The right internal jugular vein was surgically isolated and cannulated with a flexible catheter connected to a pre-calibrated infusion pump, loaded with both normal saline and saline mixed in 10% ProHance (Gadoteridol, Bracco Diagnostics Inc., Milan,)/formalin solutions. ProHance is a gadolinium chelate that significantly reduces T_1_ of cardiac tissue, thus allowing for short TR imaging with near complete T_1_ recovery between pulses. A 1.3% agarose/PBS solution was prepared and set aside. With the internal jugular venous cannula secured in place, a midline abdominal incision was made to expose the peritoneal cavity. The diaphragm was then carefully incised, dissected away from the cardiac apex, and removed. The descending thoracic aorta and inferior vena cava were exposed and isolated. The anesthetized, ventilated animals were then perfused with approximately 50 mL 0.9% NaCl over a period of 5 minutes. When perfusion began, the inferior vena cava (IVC) and descending thoracic aorta were incised at the level of diaphragmatic hiatus, allowing blood to rapidly clear from the thorax, upper extremities, and head. At the end of 5 minutes, the saline reservoir was exchanged for a 10% ProHance/formalin solution, and the animals were perfused with this solution for an additional 5 minutes, allowing for rapid fixation of the myocardium. Once perfusion fixation was complete, the animals were perfused with a heated 1.3% liquid agarose solution. Heating the solution decreases viscosity such that the solution crosses the pulmonary capillary bed and enters the left side of the heart. The animals were perfused until the gel solution could be seen emanating from the transected descending thoracic aorta. At this point, the infusion pump was switched off, and the aorta and the IVC were clamped to prevent outflow of liquid agarose from the heart. The liquid was then allowed to cool and form a semi-solid gel within the cardiac chambers, preserving anatomic atrial and ventricular shape and preventing chamber collapse. The heart was then carefully excised by transecting the great vessels and pulmonary veins, with attention to maintaining the integrity of the right and left atrial walls. The excised hearts were immersed in Fomblin® (perfluoro-polyether) (Solvay Solexis, Inc. West Deptford, NJ, USA) and immobilized in a specialized MR-compatible specimen tube.

### Diffusion tensor CMR

Imaging was performed at the Duke Center of In Vivo Microscopy (Duke University Medical Center, Durham, NC, USA) on a 9.4 T vertical-bore Oxford magnet equipped with a gradient system able to achieve peak values of 2000 mT/m. A 3D DW-SE pulse sequence was used to scan the samples with TE (11.8 ms) and TR (100 ms). The acquisition matrix was 256x256x256, resulting in an isotropic voxel size of 43 μm. Diffusion was encoded using a pair of half sinusoidal gradient lobes of 1600 mT/m in amplitude, 1.3 ms in width, and 6.8 ms in separation. One b_0_ and six diffusion weighted image stacks were acquired (in a total scan time of 12.7 hours) with an empirically chosen b value of 1852 s/mm^2^. Other imaging parameters were: Bandwidth = 62.5 kHz, FOV = 11 × 11 × 11 mm^3^ full-Fourier encoding, one average, single echo. All specimens were scanned in a 12 × 25 mm^2^ (diameter × length) solenoid radiofrequency coil.

### Image registration using LDDMM

**Figure 1:**
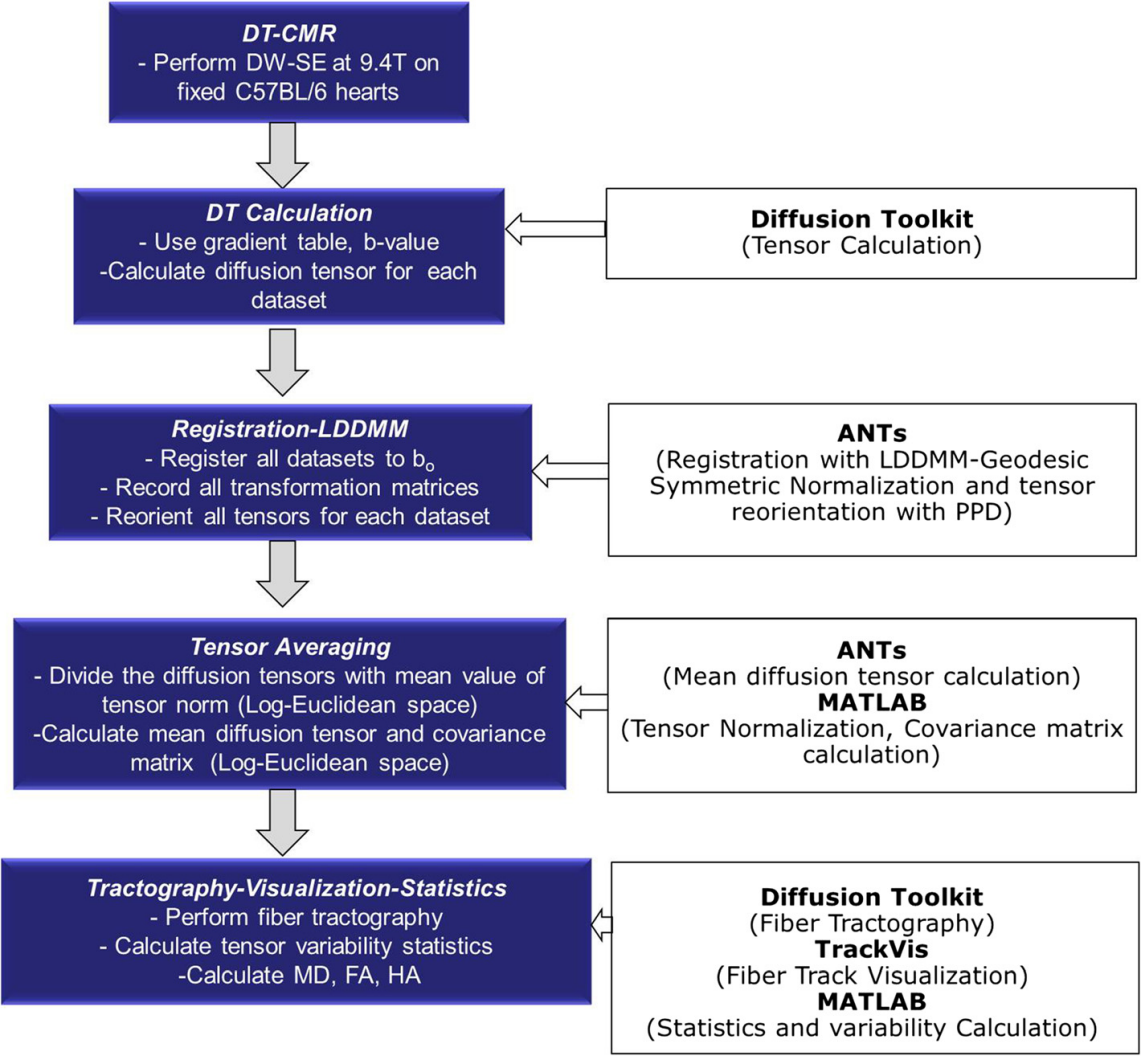
**Schematic representation of sequential data flow and processing of DTCMR.** (Left) Procedural steps for imaging acquisition and processing, and (right) software, computational algorithms and mathematical operations employed for analyses.

**Figure 2:**
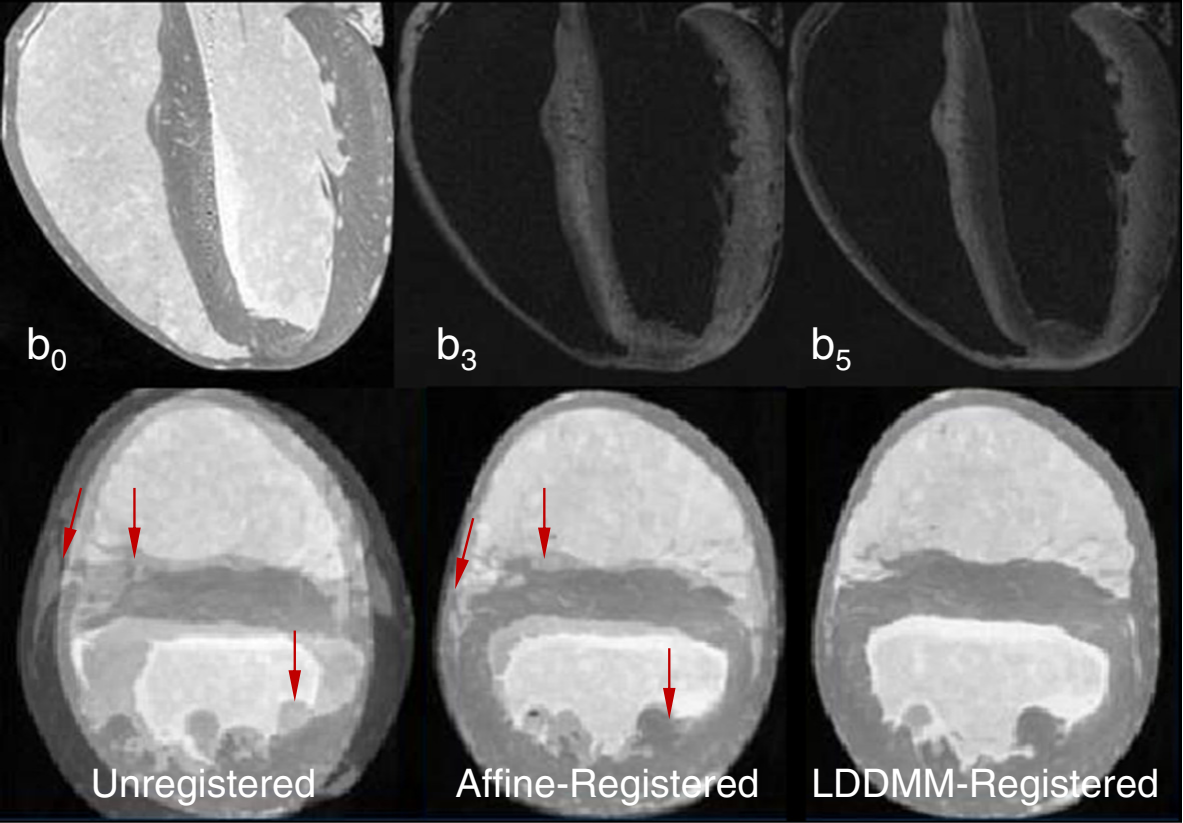
**Visualization of DTCMR and image registration.** Typical (top) long- and (bottom) short-axis b-weighted diffusion images (indicatively showing only b_0_, b_3_, and b_5_ for the six-directional encoding scheme adopted) of the ex vivo, fixed murine heart (the window/level applied was similar only for the b3- and b5-weighted images); (bottom left to right) unregistered, affine- and diffeomorphically-registered target-template images. Arrows indicate misregistration areas

**Figure 3:**
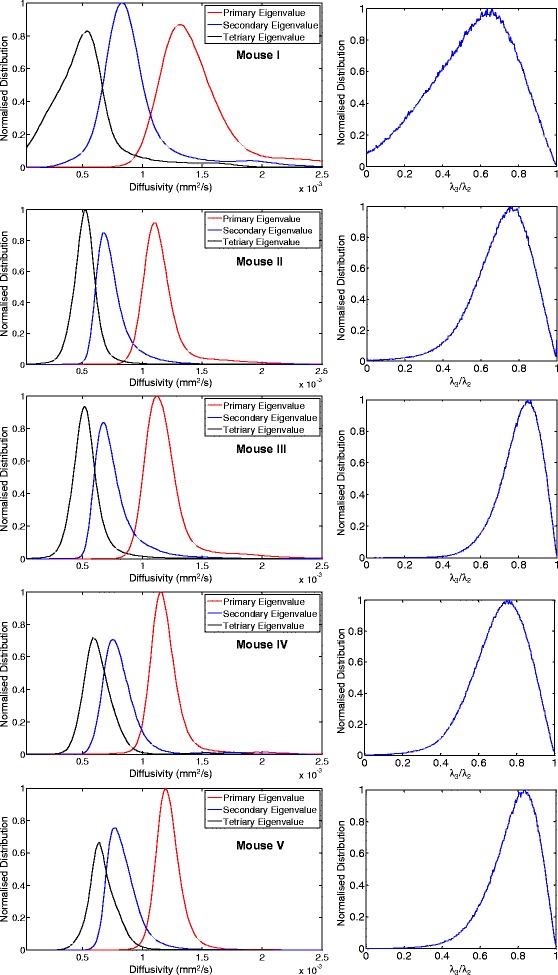
**Overview of tensor map registration and eigenvalue distributions in justification of trans-myocyte anisotropy.** Registration process in accordance to the Procrustes mean approach; (Left) typical eigenvalue histogram and (right) ratio (λ_3_/λ_2_) distributions from each of the five murine hearts used to construct the mean tensor map; indicative are the relative overlap of distributions of the unsorted secondary and tertiary principle values and the skewed ratio distributions.

*Estimation-Registrations of Diffusion Tensors:* The diffusion weighted MR images were imported into the Diffusion Toolkit (Massachusetts General Hospital, Boston, MA, USA; http://trackvis.org/dtk/) along with the corresponding gradient table and b–value, and the diffusion tensors for each dataset were calculated (Figure 1). All diffusion datasets were co-registered with the corresponding b_0_ images (Figure 2). Registration was performed using the Advanced Normalization Tools (ANTs) [38]. The geodesic mean, group-wise methodology [5,18,23,24,39] was adopted. According to this methodology, a source heart image set was chosen (template) from the population of imaged (target) datasets mapped to the template heart [5,18,39,23,24]. This process defined a new template heart, and the methodology was then iteratively repeated until an average geometry was obtained that resided within the center of all target geometries (Figure 2, 3). Specifically, Large Diffeomorphic Metric Mapping (LDDMM) [40] with Geodesic Symmetric Normalization (SyN) transformation [38] was applied to register each target dataset to the current template dataset (Figure 2).

Mutual information was defined as the similarity metric of the registered b_0_ images. The quality of the process was assessed using the union overlap measure (Jaccard coefficient) [41], by summing the myocardial voxels in the target image that matched myocardial voxels in the template image, and dividing by the total number of myocardial voxels (Figure 2). The resulting transformation matrices and deformation fields obtained from the b_0_ image registration were subsequently used to register datasets to the reference template of the heart geometry, and to reorient the diffusion tensors using the Preservation of the Principle Direction (PPD) methodology [42].

### Diffusion tensor averaging – construction of mean myocyte orientation tensor map

*Diffusion Tensor Normalization:* To account for factors responsible for diffusion tensor dispersion not associated with intrinsic variability (for example, temperature), the diffusion tensors were globally normalized. Such normalization was performed in the Log-Euclidean space of the diffusion tensors (*D*_log_), where the individual mouse datasets were divided by the mean value of the tensors’ norm $\sqrt{Tr\left({D}_{\log}^2\right)}$, accounting only for voxels within the myocardium [27,43,44].

**Figure 4:**
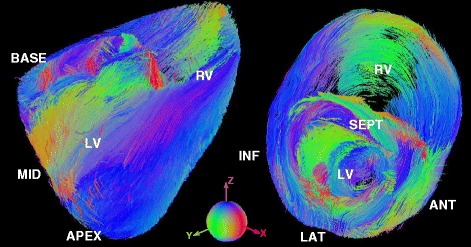
**Visualization of local myocyte/myofiber tractograpy maps.** (Left to right) Tractography of the mean diffusion tensor presented in long and short axis views using a standard XYZ → RGB directionality color-coding scheme.

*Tensor Averaging – Variability Quantification*: The registered/reoriented diffusion tensor fields were then averaged independently at each voxel using Log-Euclidean tensor averaging calculus in ANTs [38], resulting in the mean tensor (${\overline{D}}_{\log }$) (Figure 4) and the corresponding covariance matrix (Σ), in accordance to:

1 $$ {\overline{D}}_{\log }(X)= \exp \left(\frac{1}{N}{\displaystyle \sum_{i=1}^N \log \left[{D}_i(X)\right]}\right) $$

2 $$ \varSigma (X)=\frac{1}{N-1}{\displaystyle \sum_{i=1}^N\left\{vec\left[\varDelta {D}_i(X)\right].vec\left[\varDelta {D}_i{(X)}^T\right]\right\}} $$

where N is the number of studied hearts, X is the voxel position, vec (ΔD_i_) is the minimal representation of ΔD_i_= $log\left({D}_i\right)-{\overline{D}}_{log}$, and Σ is a representation of the covariance matrix in the log-space [23,24,27].

The variability of the mean diffusion tensor was assessed by projecting the covariance matrix on the orthonormal basis W_ii_ and W_ij_ (Equations 3, 4) [27]. Such orthonormal basis allowed the calculation of the eigenvalue (λ_i_) and eigenvector (v_i_) variabilities (Equations 5, 6) [27].

3 $$ {W}_{ii}={v}_i\cdot {v}_i^T $$

4 $$ {W}_{ij}=\frac{1}{\sqrt{2}}\left({v}_i\cdot {v}_j^T+{v}_j\cdot {v}_i^T\right) $$

5 $$ E\left(\delta {\lambda}_i^2\right)=vec{\left({W}_{ii}\right)}^T\cdot \varSigma \cdot vec\left({W}_{ii}\right) $$

6 $$ E\left({\varepsilon}_{ij}^2\right)=\frac{1}{2{\left({\lambda}_i-{\lambda}_j\right)}^2}vec{\left({W}_{ij}\right)}^T\cdot \varSigma \cdot vec\left({W}_{ij}\right) $$

**Figure 5:**
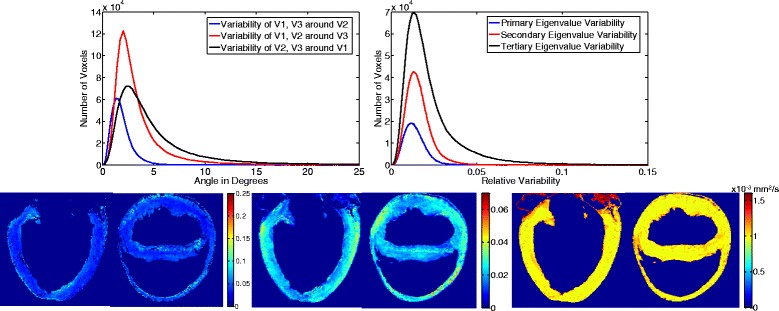
**Tensor map** - **based eigenvalue, eigenvector variability and quantitative diffusion biomarker maps.** (Top) Histograms of eigenvalue (left) and eigenvector variability distributions (right); (Bottom) Mid-ventricular long and short axis views of the (left) covariance matrix trace, (middle) FA, and (right) MD maps of the mean tensor map.

The norm $\sqrt{\;Tr\left[\varSigma (X)\right]}$ of such covariance matrix subsequently allowed estimation of the relative variability of the diffusion tensor (Figure 5), in accordance to methodology published earlier [27].

### Tractography

**Figure 6:**
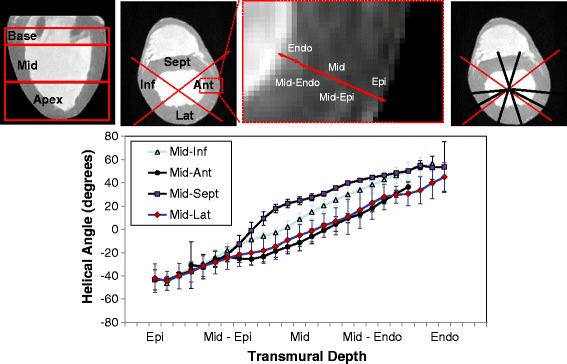
**Schematic representation of sectoral distributions for quantitative analyses and transmural distributions of helical angles (HA).** (Top) Regional myocardial segmentation identifying (left) basal, middle, and apical areas, (middle) left ventricular (LV) anterior, lateral, inferior and septal areas, and epicardial to endocardial areas (Epi, Epi-Mid, Mid-Endo, Endo); (right) sectoral representation for HA profile analyses. (Bottom) Plots of transmural helical angle trajectories in middle-myocardium, based on sectoral analyses.

The calculated mean Diffusion Tensor was then re-imported in Diffusion Toolkit where myocyte tracking was performed, employing the second-order Runge–Kutta method and using an empirically chosen angle threshold of 40° [26]. FA, MD, and HA maps were subsequently calculated, constructed, visualized (Figure 6), and quantified. Specifically, a sectorial analysis approach was endorsed (Figure 6), where the short-axis myocardium was segmented by an expert into anterior, lateral, inferior, and septal regions (in accordance to prior AHA guidelines and other publications [21,45]). Correspondingly, basal (27 slices), middle (58 slices), and apical (57 slices) areas were identified along the long-axis. While segmented short-axis areas were chosen to have similar areas, the number of basal slices analyzed was not proportional to the number of middle-myocardial and apical slices (Figure 6). Misregistration errors at the location of large vessels led to exclusion of a number of basal slices from subsequent analyses.

For HA assessment, five transmural myocardial layers were defined (each having approximately 20% of transmural thickness), that included epicardial (Epi), epicardial-middle (Epi-Mid), middle (Mid), middle-endocardial (Mid-Endo), and endocardial (Endo) regions (Figure 6). Multiple radial profiles were taken spanning sectorial regions for each of the four short-axis regions, in accordance to methodologies proposed and published previously [21,30]. Within each wedge, helical angles were transmurally measured along radial samples in 1.5° increments, over 3 consecutive slices chosen in the middle of each respective area.

### Trans-myocyte anisotropy

**Figure 7:**
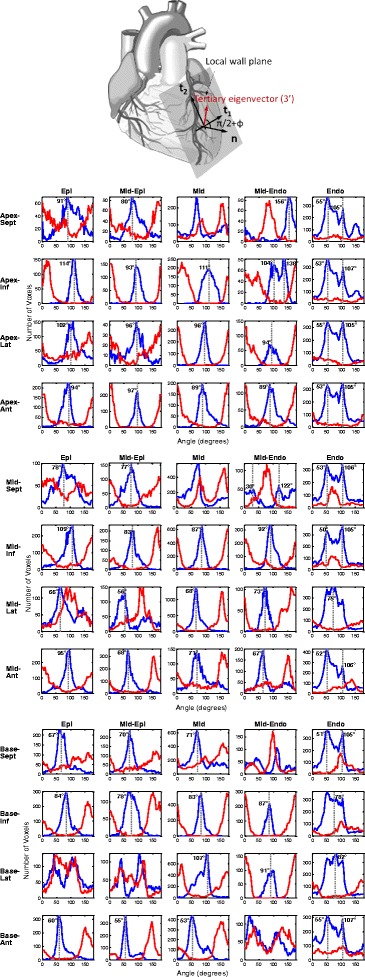
**Schematic representation of intersection angle and distributions identifying trans-myocyte anisotropy.** (Top) Schematic representation of the epicardial local wall plane with vectorial definition of plane directions (t_1_, t_2_), the unit normal ($\widehat{n}$), and the direction of the tertiary eigenvector (3΄). The intersection angle can be defined based on the angle subtended between 3΄ and the unit normal. (Bottom) Bi-modal histogram distributions identifying areas of trans-myocyte anisotropy. Superimposed (in red color) are corresponding representations of the secondary eigenvector (2΄), identifying sub-regions where the histogram distributions differ. Intersection angle distributions in areas of trans-myocyte anisotropy are supportive of presence of two dominant myocardial sheetlet layers at 62.7 ± 11.8° and 99.5 ± 11.9° for the various myocardial regions.

Differing secondary and tertiary eigenvalues and their associated ratio (λ_3_/λ_2_) distributions (Figure 3) are indicative of the existence of possible trans-myocyte structural anisotropy, regardless of eigenvalue statistical biases and susceptibility to sorting errors [7]. Cluster sectorial analyses and modal-histogram plots were generated to verify existence of dominant layers in mice (based on computation of intersection angles (φ) [20] Figure 7), for basal, middle and apical areas, for all transmural myocardial layers and sectors. Identification of dominant angles was subject to histogram peak detection based on derivative zero-crossings or cumulative integration inflexion points.

### Statistics

All results are reported as mean ± SD. Constancy of FA, MD, and linearity of HA values within short axis areas for basal, middle, and apical myocardium for the constructed tensor map is reported as between (averaged estimates for all the mice studied) coefficient of variabilities (e.g. CV = 100xSD/Mean). Non-parametric Wilcoxon, two-tailed t-tests were also employed (XLSTAT, Paris, France) to statistically compare FA, MD, and HA differences (sectorial and globally averaged values) at the 1% significance level. Assessment for the existence of significant variability for all such recorded parameters was achieved using one-way ANOVA tests (myocardial location).

For SNR measurements, four circular ROIs (having the same area of 0.23 mm^2^) were placed in the Anterior, Inferior, Lateral and Septal myocardial wall areas in transversal apical/middle/basal slices. The mean signal intensity was measured in these regions (using ImageJ, http://imagej.nih.gov/ij/, Bethesda, MD, USA) along with the standard deviation of the signal in an additional ROI positioned in the background (outside the image object, having an area of 1.84 mm^2^). The ratio between the mean intensity of the myocardial ROIs and the background SD yielded the SNR for each myocardial region. Given the large estimated SNR values, no bias correction was imposed to such estimates.

## Results

The sequential flow and data processing for the methodology adopted for the tensor map construction is summarized in Figure 1. In addition to MR imaging, the remaining steps include diffusion tensor calculation, registration, tensor re-orientation, averaging and normalization, and quantitative and statistical analyses.

For all mice included in the study, measured dimensions of the heart ranged from 7.4-7.9 mm (mean ± SD, 7.7 ± 0.2 mm) in long axis (base-apex), and 4.5–5.2 ± 0.5 mm in short axis (basal diameter). Such results closely match murine cardiac dimensions reported in the in vivo and ex vivo state [21,31]. These results also suggest the consistency in tissue fixation, allowing proper quantitative diffusion biomarker estimations and comparisons. Mean SNR values for non-weighted (b0) and fully-weighted scans (b6) scans were 58.9 ± 8.3 and 33.5 ± 7.5, respectively. Such values are dismissive of the likelihood of presence of significant noise biases [46,47] on quantitative eigenvalue and eigenvector estimations.

For the tensor map construction, iterative group-wise LDDMM registration was employed (Figures 2 and 3). Three specific diffusion weighted views, indicative of the image quality of the adopted six-encoding directional scheme are shown in Figure 2. The quantitative accuracy of registration was based on estimation of the union-overlap measure for registered-target images [Jaccard coefficient] that ranged between 88.5–93.6% (mean ± SD; 90.3 ± 2.1%).

The statistical tensor map presented here (visually displayed using tractography in long and short-axis views, as shown in Figure 4) includes reporting on both global and local population myocyte tensor variability of the principle diffusion direction (Figure 5). Disparity in the trace of the tensors is observed in the histograms shown in Figure 3. Therefore, to eliminate possible dependency on diffusion values from external factors (leading to varying scaling of the population tensors), tensor normalization was implemented.

Global diffusion tensor variability is embedded in the covariance matrix (Equation 2); analyses were thus based on the calculation of mean eigenvalues and eigenvectors, but reported as the mode of the standard deviation of the eigenvalue distributions with respect to the mean [23,24], found to be 1.2% (σ_λ1_), 1.3% (σ_λ2_), and 1.4% (σ_λ3_). Overall, the eigenvalue variability of the constructed tensor map (based on normalized tensors) is within acceptable ranges, indicative of intra-cardiac and intra-species diffusion rate homogeneity. Figure 5 also depicts variability maps for both FA and MD.

Quantitative results (Figure 6) led to mean diffusion tensor eigenvalues of λ_1_ = 1.12 ± 0.17, λ_2_ = 0.86 ± 0.17, λ_3_ = 0.69 ± 0.17 (×10^−3^) mm^2^/s, and to global mean FA and MD values of 0.25 ± 0.07 and 8.9 ± 1.6 × 10^−4^ mm^2^/s, respectively, for all hearts (before normalization) and to corresponding FA and MD values of 0.021 ± 0.01 and 1.00 ± 0.26 (after tensor normalization). Sectorial myocardial analyses yielded non-normalized and normalized FA/MD values, as shown on Table 1.

**Table 1:** **Summary of quantitative results for diffusion biomarkers and local myocyte orientation**

**Parameter (Non-normalized/normalized)**		**HA Range (°)**					**FA**					**MD (x10** ^**−3**^ **mm** ^**2**^ **/s)[Non-Normalized]** **MD (mm** ^**2**^ **/s) [Normalized]**				
**Region**		**Epi**	**Epi-Mid**	**Mid**	**Mid-Endo**	**Endo**	**Epi**	**Epi-Mid**	**Mid**	**Mid-Endo**	**Endo**	**Epi**	**Epi-Mid**	**Mid**	**Mid-Endo**	**Endo**
**Apex**	**Septal**	19 ± 8	31 ± 2	37 ± 2	39 ± 1	26 ± 9	0.234/0.019	0.251/0.020	0.260/0.021	0.244/0.020	0.210/0.017	0.862/0.998	0.843/0.989	0.829/0.982	0.836/0.990	0.845/0.993
	**Inferior**	−10 ± 3	2 ± 8	25 ± 7	41 ± 4	44 ± 3	0.307/0.026	0.312/0.026	0.275/0.022	0.240/0.020	0.224/0.018	0.812/0.980	0.807/0.976	0.829/0.981	0.837/0.977	0.862/0.984
	**Lateral**	−50 ± 8	−32 ± 4	−13 ± 8	18 ± 8	40 ± 7	0.225/0.018	0.241/0.019	0.235/0.019	0.251/0.020	0.241/0.019	0.835/0.995	0.810/0.980	0.837/0.982	0.876/0.992	0.935/1.033
	**Anterior**	−18 ± 12	−20 ± 3	−8 ± 4	7 ± 6	27 ± 6	0.224/0.019	0.303/0.025	0.269/0.021	0.251/0.020	0.241/0.019	0.992/1.016	0.866/0.992	0.887/0.996	0.901/0.993	0.918/1.002
**Mid**	**Septal**	−39 ± 5	−11 ± 15	25 ± 5	42 ± 4	52 ± 5	0.210/0.017	0.226/0.018	0.261/0.021	0.269/0.022	0.227/0.018	0.868/0.990	0.832/0.982	0.800/0.977	0.816/0.989	0.859/1.000
	**Inferior**	−37 ± 7	−14 ± 7	9 ± 9	34 ± 7	52 ± 5	0.308/0.025	0.347/0.029	0.312/0.026	0.258/0.021	0.228/0.019	0.805/1.026	0.783/0.989	0.807/0.982	0.823/0.977	0.832/0.981
	**Lateral**	−41 ± 3	−24 ± 5	−3 ± 8	23 ± 8	39 ± 6	0.266/0.022	0.294/0.025	0.255/0.021	0.221/0.019	0.204/0.016	0.845/1.020	0.790/0.983	0.812/0.978	0.832/0.989	0.908/1.049
	**Anterior**	−28 ± 4	−23 ± 3	−11 ± 5	7 ± 6	28 ± 8	0.266/0.021	0.322/0.026	0.296/0.024	0.281/0.022	0.249/0.020	0.932/1.022	0.853/0.987	0.850/0.984	0.843/1.000	0.883/1.013
**Base**	**Septal**	−47 ± 2	−25 ± 13	13 ± 11	37 ± 5	54 ± 5	0.227/0.018	0.231/0.019	0.250/0.020	0.275/0.023	0.243/0.020	0.851/0.987	0.807/0.977	0.768/0.973	0.757/0.969	0.779/0.972
	**Inferior**	−43 ± 7	−25 ± 6	−7 ± 6	15 ± 8	41 ± 8	0.304/0.025	0.368/0.031	0.342/0.028	0.269/0.022	0.227/0.019	0.820/1.030	0.766/0.995	0.804/0.989	0.828/0.977	0.848/0.990
	**Lateral**	−49 ± 3	−40 ± 3	−22 ± 8	−3 ± 5	15 ± 5	0.323/0.028	0.310/0.026	0.236/0.020	0.214/0.018	0.212/0.017	0.804/0.999	0.804/0.986	0.813/0.978	0.822/0.981	0.840/0.992
	**Anterior**	−20 ± 6	−30 ± 2	−28 ± 2	−19 ± 5	−5 ± 5	0.251/0.020	0.306/0.026	0.273/0.022	0.243/0.020	0.204/0.017	0.934/1.075	0.821/0.990	0.803/0.975	0.801/0.984	0.833/1.017
**Globally averaged value** ^*^		**111 ± 23**	**[n = 5, C57BL/6;b = 1850]**		**(This study)**		**0.25 ± 0.07 (This study)**					**0.89 ± 0.16 (This study) [Non-normalized]**				
		126 ± 3*	[n = 10, 129/ola]		(Healy 2011)		0.27 ± 0.06 [33]					0.75 ± 0.13 [33]				
		143 ± 12*	[n = 2, 129/ola;b = 1130]		[33]		0.38 ± 0.16 [55]					0.7 ± 0.25 [55]				
		110	[n = 14,C57B16/J]		[49]											
		125[55]	[n = 4, Swiss;b = 1440]		[55]											

Sectorial (non-normalized normalized) and globally-averaged quantitative comparison of DTI-based parameters (HA, FA, MD) from the constructed mean tensor map and based on fixed murine hearts and the PPD re-orientation/registrations strategies.

*Transmurally-averaged values (epicardium to endocardium) from the entire LV myocardium (including anterior, lateral, inferior and septal areas).

Sectorial statistical quantitative analyses resulted in CV values that ranged between (non-normalized/normalized) 3.8–15.8%/2.4–11.1% for MD and 14.7–32.0%/16.6–37.4% for FA. Higher CV values resulted in lower statistical power in biomarker estimation.

Statistical t-tests showed insignificant differences of mean normalized FA and MD for different sectors at the three cardiac levels [Basal-Middle-Apical]. Additionally, ANOVA results indicate no significant variability for FA and MD in all myocardial areas, in justification of homogeneity of distribution of reported values. Such findings demonstrate that diffusion properties are similar among the normal population of studied mice.

Diffusion orientation is associated with differing variability along each of the three principle directions, manifested as a measure of diffusional coherence along the three orthonormal directions [23,24,27]. Local myocyte orientation variability in longitudinal and equatorial planes is reported by modes σ_θ1,3_ and σ_θ1,2_; σ_θ2,3_ reports the laminar structure variability. Specifically, the standard deviations exemplifying variability of rotational differences of eigenvector pairs with the third orthonormal (based on the arctan of Equation 6) [23,24], are found to be 2.0° (σ_θ1,2_), 1.4° (σ_θ1,3_), 2.6° (σ_θ2,3_), respectively. Local myocyte orientation variability has also been traditionally analyzed by the transmural distribution of the HA. These are reported to range between −57° and +60° with typical sectorial transmural trajectories depicted on Figure 6. We found a correlated dependency of HA with transmural depth, with Figure 6 demonstrating a counter-clockwise epicardial to endocardial sectorial HA variation in all short-axis planes. The globally estimated HA exhibited a mean transmural variation of 111 ± 23°, in close agreement with prior studies [4] (Table 1).

Significant and extensive evidence from previous work [1-3] supports the aggregation and organization of cardiac myofibers in sheetlets with spatially varying orientation. Theoretical and histological reconstructions have predicted and verified the existence of two distinct sheetlet populations in canine [48], sheep [34], and ovine ventricles [49]. Initial evidence for existence of trans-myocyte anisotropy (Figure 3) was therefore reinforced by the results of Figure 7, showing analyses of intersection angles based on tertiary eigenvector orientation, indicative of a distribution exhibiting sheetlet dominance—in agreement with earlier studies on canines [19].

## Discussion

The importance of quantitative DTCMR microscopy atlases and maps to the study of cardiac structure and function has been defined previously [4]. Nevertheless, only two comprehensive cardiac atlas attempts have been documented, on canines [27] and humans [22-24]. Thus, this effort introduces, to the best of our knowledge, the first integrated, quantitative cardiac tensor map of the C57BL/6 mouse, one of the most extensively studied and stable murine strains. Compared to previous publications on DTCMR on the same mouse strain by Jiang et al. [4] and Schmit et al. [31], this study is unique in that it presents isotropic data at 43-μm resolution, and extends quantitative analyses beyond global eigenvalue estimation and HA variability [4,31] to include FA, MD, myofiber and laminar sheetlet variability, as well as trans-myocyte anisotropy.

For these reasons, this population-based tensor map defines a crucial juncture in achieving our long-term goal of establishing a common reference for future phenotypic studies. The tensor map can be used in several ways to help identify (either on a voxel-based or sectorial-based approach) common local murine myocyte architectural features and population-based variabilities for quantitative (normalized and non-normalized) comparisons, in both pathologic and transgenetic states. Additionally, it can serve as a template reference for inter- and intra-species comparisons, addressing questions about structural-functional conservation and embryonic-adult development.

Ventricular myofiber geometry and orientations were initially studied based on histological reconstructions pioneered by Nielsen et al. [50], with a maximum attained measurement resolution of 500 μm. DTCMR emerged and was established as a non-invasive method to surpass Nielsen’s approach, attaining interspecies, in-plane spatial resolutions that ranged from 78–400 μm [4,12,19,33].

While limited success has been evidenced thus far in in vivo animal [14] and human DTCMR studies [16], the vast majority of prior attempts focused on ex vivo protocols, mostly conducted under formalin fixation of tissue. For example, using light microscopy, Grimm et al. [51] quantified average sarcomeric shrinkage of 4.2% in rat papillary muscle, upon formalin fixation. Such results have been consistently supported by recent DTCMR reports indicating minimal formalin fixation effects on primary, secondary and tertiary eigenvalues [4,12] and correlation of these findings with invasive histology [33].

Basic geometric LV measurements reported in this study also dismiss possible formalin-based fixation effects. They also support consistency in inducing fixation at the end-diastolic conformational state. Additionally, the adopted six-directional encoding scheme is in compliance with the realm of a proper acquisition [52-54], yielding comparatively adequately high SNR values [4,21], dismissing possible biomarker noise bias effects, with anticipated myofiber orientation accuracy of approximately 6°, on the order of Jiang’s study [4]. Possible errors arising due to the adopted encoding scheme are compensated somewhat by the high SNR and the increased spatial resolution of the ex vivo DT MRI [42,55,56].

Methodologically the adopted data registration schemes, based on LDDMM techniques [19], allow precise voxel and sectorial analyses, preserving the spatial variability of myofiber architecture. Although evaluation of all possible tensor reorientation strategies is beyond the scope of this work, DTI analyses yielded consistent mappings of scalar measures.

Similarly, patterns of eigenvalue and λ_3_/λ_2_ distributions highly agree with prior canine [27] and human [22,24] atlases, indicating trans-myocyte anisotropy. However, the reported σ_λi_ values (1.2, 1.3, and 1.4%) are by far smaller and less variable than canine (5.35, 6.35, and 8.69%). Of course, care must be exercised in interpreting differences between human, canine, and murine data (especially angular dispersions of tensor eigenvectors), given the disparity of acquired resolutions, field-of-views and the relative cardiac scale sizes of the three species. Regardless, in both human and canine species the pattern seems to be conserved, with the primary eigenvalue exhibiting lesser variability compared to λ_2_ and λ_3_. It is highly likely that the primary source of observed increased variability of λ2 and λ3 is attributed to the intrinsically lower signal associated with the respective diffusional processes.

Global tensor variability of 4.98 ± 1.6% compares well with reported values of 10% and 13.2% in canine [27] and humans [23,24], respectively, supporting structural stability. Variabilities of myofiber orientation (assessed by angular dispersion patterns of the primary and secondary, and tertiary and primary eigenvector pairs over their orthotropic eigenvector counterparts, v_3_ and v_2_, respectively) are reported to be 2.0° and 1.4°, respectively, in comparison to 7.9° /7.7° and 13.0° /11.5° in canine and humans [23,24,27].

Laminar sheetlet orientation variability (assessed through v_2_ and v_3_ orientation about v_1_) seems to be less stable at 2.6°. Such pattern is in agreement with findings in canine and humans, in support of the loosely organized laminar sheetlet structure. Nevertheless, the murine heart seems to be highly structured and stable, exceedingly so when compared to the canine (22.7°) and humans (31.1°). The smaller global tensor variabilities observed in the mouse may be physiologic, with the small-sized murine heart having evolved a less variable structure (compared to other species), but also may likely be attributed in part to the high-resolution of the acquired data. At an isotropic resolution of 43 μm^3^, and a typical cardiac myocyte phenotype of column-shaped cells of approximately 50–150 μm in length and approximately 20 μm in width, imaging is representative of only very few cells.

The short- and long-axis maps of the norm of the covariance trace of Figure 5 indicate that although certain septal and basal areas exhibit higher spatial variability and heterogeneity across the LV, the myocardium retains a highly organized structure. Similar findings are evident for MD. However, increased spatial dispersion is documented for FA.

In addition to spatial variability, global and sectorial values (normalized and non-normalized) for HA, FA and MD are shown on Table 1. While close agreement of global statistics for all such indices is recorded against prior studies [4,21,31,32], increased sectorial CV was associated with FA. Overall, mean quantitative (non-normalized) FA results concur with prior studies reporting values ranging from 0.1–0.6 [29] for various species, and exhibiting a mean value of 0.27 ± 0.06 for the murine C57BL/6 myocardium [4].

Additionally, the HA distribution of local myocyte orientation has been studied and quantified across myocardial segments. Both the sectorial pattern of transmural helical angles (HA) range and absolute values match those of Healy et al. [21], as well as the reported values in other studies [4,12,31]. The HA spans from −57° – +23° on the epicardium to +15°– +60° on the endocardium, with a noted correlation with transmural depth. Sectorial plots of transmural HA exhibit, however, an increased variability as reflected by the increased SD values (that ranged between various transmural locations ranged from 2–15°). Such variation is a composite reflection of anatomical variability, measurement noise, and registration errors.

Sheetlet dominance is confirmed at mean angles values of 62.7 (±11.8) ° and 99.5 (±11.9) °, in comparison to reports by Helm et al. [20] reporting angles at 35° and 115° and a mean difference of 78° in canines. Dominant muscle layers were also identified previously by numerous studies, including Dokos et al. [57], Arts et al. [48], both reporting angles at 45° and 135°. Such results are consistent with the sliding sheetlet hypothesis, showing that the laminar structure is comprised of two primary orientation populations [35,58,59].

## Conclusions

Quantitative murine myocardial tractography at an isotropic resolution of 43-μm of the ex-vivo heart was accomplished in approximately 12 hours of data acquisition, sensitized to 6 diffusional directions. Fiber tractography yielded local myocyte orientation maps associated with mean fractional anisotropy (FA) of 0.25 and mean mid-ventricular HA distributions that ranged between −41° – +52°, in agreement with prior published results in humans and canines. This mouse statistical tensor map is the first of its kind in that it quantitatively estimates both normalized and non-normalized diffusion biomarkers. We believe that the tensor map will be of value in the process of phenotyping pathological and transgenetic cardiac states.

## Glossary of terms

**CMR:** Cardiovascular magnetic resonance

**DTI:** Diffusion tensor imaging

**Fractional anisotropy (FA):** Scalar value between zero and one that describes the degree of anisotropy of a diffusion process (i.e. the degree of motional restriction along specific directions). A value of zero means that diffusion is isotropic, i.e. it is unrestricted (or equally restricted) in all directions. A value of one means that diffusion occurs only along one axis and it is fully restricted along all other directions.

**Helix (Helical) angle (HA):** The angle of obliquity of the principal eigenvector relative to the local short axis plane or relative to local circumferential direction, if necessary, projected radially onto the local wall tangent plane. Positive and negative values can be used for clockwise or anticlockwise rotation, for example as viewed from the lumen outward.

**Intersection angle:** The angle formed between the local wall plane unit normal ($\widehat{n}$) and the projection of the tertiary eigenvector onto the local wall plane (defined by vectors t2 and n).

**Laminar structure:** An inclusive alternative to ‘sheetlet and shear layer’ structure.

**Mean Diffusivity (MD):** The trace of the diffusion tensor; it is a scalar measure of the total diffusion within a voxel.

**Myocyte:** Basic contractual cell unit of myocardium composed of (among others) myofibers, serially connected sarcomeres via gap-junctions.

**Shear layers:** Fissures or interstices between adjacent sheetlets. They are thought to allow adjacent sheetlets to change imbrigation angle and shear relative to one another during contractile function.

**Sheetlet:** Aggregate arrangements of a few cardiac myocytes in planes having microscopic thicknesses and limited spatial extent.

**Tractography:** Visualization of distributed eigenvector orientations by plotting virtual tracts that, at all points along them, are aligned with local eigenvectors.

**Transmural angle:** The angle of transmural obliquity, for example of the 2nd eigenvector of diffusion (or of laminar structures visualized directly by microscopy) relative to the local wall tangent plane, measured in the plane perpendicular to the 1st eigenvector direction (or the local myocytes).

## Abbreviations

AHA: American Heart Association
ANOVA: Analysis-of-variance
DTI: Diffusion tensor imaging
DWSE: Diffusion weighted spin echo
FA: Fractional anisotropy
FS: Finite strain
HA: Helical (or Helix) angle
IVC: Inferior vena cava
LDDMM: Large diffeomorphic metric mapping
MD: Mean diffusivity
MRI: Magnetic resonance imaging
PBS: Phosphate buffered solution
PPD: Preservation of principal direction
SD: Standard deviation
SNR: Signal-to-noise ratio
SyN: Geodesic symmetric normalization
